# Inhibition of Corticosteroid-Binding Globulin Gene Expression by Glucocorticoids Involves C/EBPβ

**DOI:** 10.1371/journal.pone.0110702

**Published:** 2014-10-21

**Authors:** Nicolette Verhoog, Fatima Allie-Reid, Wim Vanden Berghe, Carine Smith, Guy Haegeman, Janet Hapgood, Ann Louw

**Affiliations:** 1 Department of Biochemistry, Stellenbosch University, Matieland, Western Cape, South Africa; 2 PPES, Department of Biomedical Sciences, University of Antwerp, Antwerp, Belgium; 3 LEGEST, University of Ghent, Ghent, Belgium; 4 Dept of Physiological Sciences, Stellenbosch University, Matieland, Western Cape, South Africa; 5 Department of Molecular and Cell Biology, University of Cape Town, Rondebosch, Western Cape, South Africa; Shanghai Jiaotong University School of Medicine, China

## Abstract

Corticosteroid-binding globulin (CBG), a negative acute phase protein produced primarily in the liver, is responsible for the transport of glucocorticoids (GCs). It also modulates the bioavailability of GCs, as only free or unbound steroids are biologically active. Fluctuations in CBG levels therefore can directly affect GC bioavailability. This study investigates the molecular mechanism whereby GCs inhibit the expression of CBG. GCs regulate gene expression *via* the glucocorticoid receptor (GR), which either directly binds to DNA or acts indirectly via tethering to other DNA-bound transcription factors. Although no GC-response elements (GRE) are present in the *Cbg* promoter, putative binding sites for C/EBPβ, able to tether to the GR, as well as HNF3α involved in GR signaling, are present. C/EBPβ, but not HNF3α, was identified as an important mediator of DEX-mediated inhibition of *Cbg* promoter activity by using specific deletion and mutant promoter reporter constructs of *Cbg*. Furthermore, knockdown of C/EBPβ protein expression reduced DEX-induced repression of CBG mRNA, confirming C/EBPβ’s involvement in GC-mediated CBG repression. Chromatin immunoprecipitation (ChIP) after DEX treatment indicated increased co-recruitment of C/EBPβ and GR to the *Cbg* promoter, while C/EBPβ knockdown prevented GR recruitment. Together, the results suggest that DEX repression of CBG involves tethering of the GR to C/EBPβ.

## Introduction

Corticosteroid-binding globulin (CBG), also referred to as transcortin or SerpinA6, is produced and secreted primarily by hepatocytes in the liver and is considered a negative acute phase protein (APP) [1], [2]. It contains a single binding site for glucocorticoids (GCs) and progesterone, both of which bind with high affinity, with an estimated 80–90% of endogenous GCs bound to CBG [3]–[6]. Although its main function is to transport and modulate the bioavailability of these steroids, the role of CBG is believed to extend to more than a carrier protein. It has been proposed that CBG acts as a reservoir for GCs and directly transports and releases these steroid hormones at target tissues during inflammation [4], [7]–[9].

According to the free hormone hypothesis, only the free fraction of steroid hormone is biologically active and able to diffuse across the plasma membrane of target tissues [10]–[12]. The ratio between free and bound steroids depends on the number of binding sites (concentration of plasma CBG) and the affinity (K_d_) for the binding sites. This implies that any changes in the levels of CBG would modify the distribution of steroids to target tissues [4], [13] and indeed, free corticosterone levels in CBG (−/−) knockout mice have been reported to be 10-times higher than in wild type mice [8].

Several factors influence CBG production including a variety of stressors and hormones [13]–[17]. GCs are the major hormone secreted during stress and they mediate their biological effect through binding to the glucocorticoid receptor (GR) whereby it is able to modulate gene expression [18]. GCs also regulate the level of their transport protein, CBG, in a negative feedback loop [4]. In humans, plasma levels of CBG are suppressed during prolonged exposure to GCs, whether endogenous, as in Cushing’s syndrome [19], or exogenous, as during administration of synthetic GCs [20]. A number of studies in rats also indicate that physiological and physical stressors down-regulate CBG production [21], [22]. Furthermore, the dramatic fall of CBG levels during stress, with concomitant substantial (2 to 20-fold) increases in free GC levels, merits its classification as a negative APP [1], [2]. In humans, for example, CBG levels are dramatically decreased during inflammation and this drastic decrease in CBG levels has been associated with impaired immune function [1], [23], [24].

**Figure 1:**
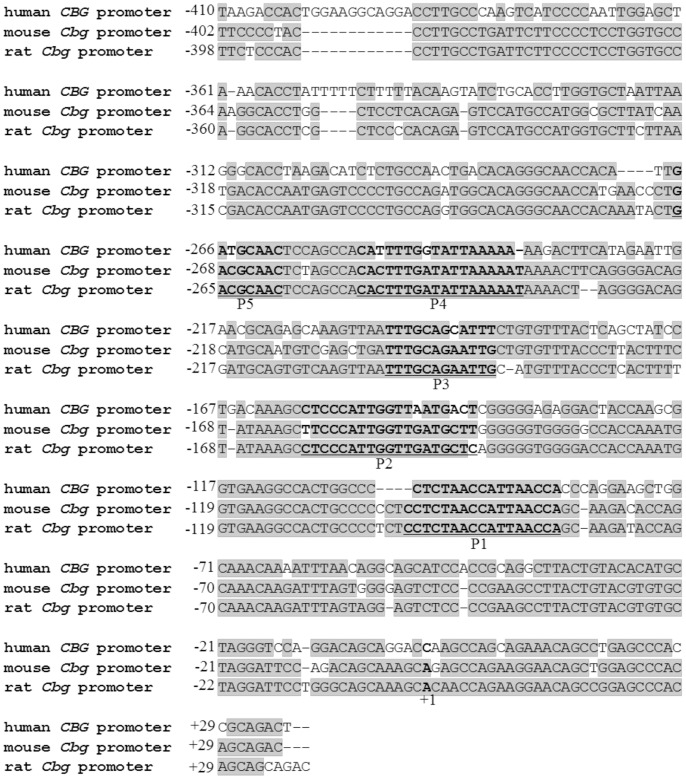
Sequence alignment of the human, mouse, and rat *Cbg* proximal promoter sequences. The human *Cbg* promoter from −410 to +29 bp (SERPINA6 ENSG00000170099), the mouse *Cbg* promoter from −402 to +29 bp (Serpina6 ENSMUSG00000060807), and the rat *Cbg* promoter from −398 to +29 bp (Serpina6 ENSRNOG00000009438) relative to the transcription start site (+1) were aligned using Bioedit. Sequences were obtained from Ensembl. The regions P1–P5, previously identified by DNase I foot printing within the rat *Cbg* promoter (25), are denoted as bold and underlined letters with P1–P5 and resemble recognition sequences for HNF1, CP-2, DBP, HNF3α, and C/EBPβ, respectively. Conserved residues, relative to the rat *Cbg* promoter, are presented as shaded.

Despite the fact that the human, mouse and rat CBG genes (*Cbg*) have been cloned, no regulatory studies to identify possible *cis*-acting sequence elements involved in GC-mediated regulation of CBG have been done. [25]–[27]. However, DNase I foot-printing of the rat *Cbg* proximal promoter, has identified five protein-binding sites (P1–P5) within −236 bp from the transcription start site in rat liver nuclear extract [25]. These five protein-binding sites are also highly conserved in the human and mouse *Cbg* gene (Figure 1) [28]. They resemble recognition sequences for hepatocyte nuclear factor-1 beta (HNF1β; P1), CCAAT-binding protein-2 (CP-2; P2), D-site-binding protein (DBP; P3), hepatocyte nuclear factor-3 alpha (HNF3α; P4) and CAAT/enhancer binding protein beta (C/EBPβ or NF/IL6; P5), respectively (Figure 1) [25]. Electrophoretic mobility shift assays (EMSAs) have confirmed that footprint one (P1) binds to HNF1β and footprint two (P2) binds to CP2 [29].

Although the molecular mechanism by which GCs influence CBG levels is unclear, the *Cbg* promoter has been shown to be transcriptionally regulated via the GR [30]–[32]. However, while the *Cbg* promoter is modulated by GCs [30], [32], [33], no glucocorticoid response elements (GREs) seem to be present in the mouse, rat or human CBG gene proximal promoters [25], [28], suggesting tethering of the GR to other transcription factors rather than binding directly to DNA. Footprints P3–P5 in the *Cbg* promoter resemble recognition sequences for DBP, HNF3α and C/EBPβ or NF-IL6 [25], and, although binding of these transcription factors has not been confirmed, it is interesting to note that HNF3α and C/EBPβ have been reported to be important in GR-mediated signaling [34], [35]. Specifically, protein-protein interaction of the GR with C/EBPβ has been reported [35] and both C/EBPβ and HNF3α are considered pioneer transcription factors as they increase chromatin accessibility at GC-responsive genes, thereby facilitating GR interaction with GC-responsive promoters [36], [37].

In this study, the molecular mechanism of action of GC-mediated repression of CBG expression was investigated. Because no consensus GR binding sites have been identified within the CBG gene promoter, delineation of GR responsiveness within the proximal rat CBG gene promoter was performed. This work identified the region between −295 and −145bp 5′ of the transcription start, which contains putative binding sites for C/EBPβ, HNF3α and DBP, as being important for GC responsiveness. Mutation of these transcription factor-binding sites narrowed down the site for GC responsiveness to the C/EBPβ binding site. Knockdown of C/EBPβ with siRNA corroborated the requirement of C/EBPβ for GC-induced repression of CBG, while ChIP and re-ChIP experiments confirmed that both GR and C/EBPβ bind concomitantly to the *Cbg* promoter and that GR-binding is attenuated when C/EBPβ is ablated. This study is the first to report on the molecular mechanism of how GCs regulate the expression of their carrier protein, CBG.

## Materials and Methods

### Test compounds and tissue culture

Dexamethasone (DEX) and mifepristone (RU486) were obtained from Sigma-Aldrich. Steroids were dissolved and diluted in ethanol. Concentration of ethanol (vehicle) added to cells was 0.1%. Dulbecco’s modified eagle medium (DMEM) and penicillin-streptomycin (Penstrep) solution were purchased from Gibco and fetal bovine serum (FBS) from Sterilab or Adcock Ingram. L-glutamine was purchased from Sigma-Aldrich. The mouse hepatoma (BWTG3, a kind gift from Guy Haegeman, University of Gent, Belgium [32]), human hepatoma (HepG2, obtained from the Medical research council, Tygerberg, South Africa who obtained it from ATCC - product number HB-8065D) and monkey kidney (COS-7, received from Guy Haegeman, University of Gent, Belgium who obtained it from ATCC - product number CRL-1651) cell-lines were grown in Falcon T150 or T75 flasks at 37°C in a 5% CO_2_ atmosphere, in DMEM supplemented with 10% FBS, Penstrep (40 U/ml) and L-glutamine (30 g/l).

### Plasmids

The rat GRα (pSVGR1) expression vector, which was kindly provided by S. Okret (Dept. of Medical Nutrition, Karolinska Institute, Sweden), was constructed as described in Miesfeld *et* *al*., 1986 (UCSF) and contains the SV40 enhancer/early promoter region, the coding region of the rat GRα cDNA and the SV40 polyA region [38]. Various rat Cbg-luciferase reporter plasmids were constructed by G.L. Hammond (London regional Cancer Centre, London, Canada) of which the rat Cbg-295Luc construct contains the five protein-binding sites, identified by DNase I foot printing [25]. The β-gal reporter plasmid (pPGKβGopbA), constitutively expressing a neomycin-resistant/β-galactosidase fusion protein under the control of the pPGK promoter from the mouse housekeeping enzyme, 3-phosphoglycerate kinase, was a gift from Dr P. Soriano (Fred Hutchinson Cancer Research Centre, Seattle, WA). Weihua Xiao (NCI-Frederick Cancer Research and Development center, Frederick, USA) kindly provided the CAAT/enhancer binding protein (C/EBPβ) expression construct.

### Ethics Statement

The Animal Research Ethics committee of Subcommittee B at Stellenbosch University ethically approved rat studies prior to initiation of experimental procedures (reference CS29/022000). All efforts were made to minimize suffering.

### Rat studies

Forty adult male Wistar rats were divided into four groups of ten rats each. Group 1 (Control) acted as the control sedentary group and underwent no intervention treatment. Group 2 (Run) was housed in rat wheels designed for this purpose and allowed to run at will and subjected to voluntary exercise stress only. Group 3 (Swim) was subjected to an involuntary swimming exercise of one hour per day for the duration of the experiment (10 days). Group 4 (Restraint) was subjected to restraint for one hour per day in small Perspex cages (8×11×17 cm) for the 10 days of the experiment. Rats were fed rat chow and water *ad libitum*, and were housed in groups of three or four in standard rat cages, except for the RUN group, which was housed individually in specially designed rat wheels. The rats were subjected to a 12 hr light-dark cycle and weighed daily. Since rats are nocturnal animals, all interventions were carried out in the late afternoon. All rats were sacrificed by decapitation on day 11 of the protocol at noon, to counter diurnal changes in endocrine measures. The livers were removed and total ribonucleic acid (RNA) was immediately extracted according to the trizol method as described by the manufacturer (Sigma). After extraction, the final RNA pellet was dissolved in 50 µl formazol (Molecular research center, Inc) and kept at –80°C until used. The RNA concentration was determined by reading the absorbance at 260 nm. CBG mRNA expression was quantified using Northern blotting as described below.

### CBG mRNA and protein expression determination

For quantification of CBG mRNA expression in rat livers, Northern blotting was done. Total RNA (20 µg) was loaded and run on a 1% formaldehyde-agarose gel and transferred to a nylon membrane (Hybond-N^+^, Amersham). To check RNA preparation integrity, EtBr staining was used to demonstrate the presence of intact 18S and 28S ribosomal bands. RNA was fixed on the membrane by using a UV crosslinking for 12 s. The membrane was prehybridized in a hybridization oven at 50°C for 1 hr with prewarmed Dig easy Hyb solution (Roche). Plasmids carrying human Cbg complementary DNAs (cDNA kindly provided by G.L. Hammond) were amplified in a DH5α-competent Escherichia coli strain and digested with EcoRI. Cbg inserts were isolated by agarose gel electrophoresis. The expected Cbg cDNA of 1.2 kb was obtained. Hybridization was performed overnight at 50°C with [^32^P]-Cbg cDNA probes labeled with [α-^32^P] deoxycytidine triphosphate using the random priming technique (Amersham megaprime labeling kit). Membranes were washed twice for 5 min in 2xSSC, 0.1% SDS at room temperature, followed by two washings for 15 min in 0.1xSSC, 0.1% SDS at 50°C. Membranes were wrapped in cling wrap and underwent autoradiography for between 24- and 48 hrs at –70°C. Autoradiograms were scanned using the densitometry program UN-SCAN-IT. Membranes were stripped using a hot 0.5% SDS solution and reprobed with [^32^P]β–actin cDNA (provided by H. Okayama).

For CBG mRNA analysis in both BWTG3 and HepG2 cells, cells were plated at a density of 1×10^5^ cells/well in a 12-well plate, treated with vehicle control (01% EtOH) or 1 nM DEX for 8 hrs, after which total RNA was isolated using TRI Reagent (Sigma-Aldrich) as per the manufacture’s protocol. The integrity of the 28S and 18S ribosomal bands was confirmed on denaturing formaldehyde agarose gels.

Total RNA (1 µg) was reverse transcribed using the ImProm-II Reverse Transcription System cDNA synthesis kit (Promega). Subsequently, semi-quantitative real time PCR was performed using the Kapa SYBR green (Kapa) and the Corbett real-time PCR machine or StepOne Plus PCR instrument (Applied Biosystems). Human CBG gene expression was measured using the following primer pair: h*CBG* (FWD) 5′–GAACTACGTGGGCAATGGGA-3′ and h*CBG* (REV) 5′-CCTGCGGACCACCTGTTAAT-3′. Mouse CBG gene expression was measured using the following primer set: m*Cbg* (FWD) 5′-AGG CTGTCACTGATGAGGAT-3′and m*Cbg* (REV) 5′-CTGAACTATCCAGGTCTGAG-3′. Expression of 18S was used as internal control in HepG2 cells and measured with the following primer pair: h18S (FWD) 5′-GAGAAACGGCTACCACATCCAAG −3′ and h18S (REV) 5′- CCTCCAATGGATCCTCGTTA −3′. GAPDH served as an internal control in BWTG3 cells and expression was measured using the following primer set (FWD) 5′- GTCCATGCCATCACTGCCA-3′, GAPDH (REV) 5′ GGCATCGAAGGTGGAAGAG-3′. Melting curve analysis was performed to confirm amplification of a single product in each sample. Relative transcript levels were calculated using the method described by Pfaffl *et al.,* 2001 [39], and were normalized to the relative 18S (HepG2 cells) or GAPDH (BWTG3 cells) transcript levels.

For the determination of CBG protein expression, cell lysates were subjected to Western blot analysis using either a mouse CBG-specific antibody (SerpinA6 (T-15) sc-79063 from Santa Cruz Biotechnology) or the mouse or human CBG-specific antibody (ab107368 from Abcam).

### Rat *Cbg* promoter reporter studies

For transient transfections 5×10^4^ BWTG3 cells/well were plated in DMEM supplemented with culture medium in 24-well tissue culture plates. Cells were transfected 24 hrs later using Fugene6 transfection reagent (3 µl/well) as described by the manufacturer (Roche). All promoter reporter constructs (r*Cbg*295Luc, r*Cbg*145Luc or r*Cbg*1200Luc) were transfected using 360 ng of Luc reporter plasmid with or without 200 ng of rat (pSVGR1), as indicated in the figure legends. In addition, 40 ng of β–galactosidase reporter plasmid (pPGKβGopbA) was included in most samples as internal standard for transfection efficiency. If β–galactosidase was not used for normalization of luciferase values, these were normalized to total protein [40]. Cells were treated for 24 hrs with different concentrations of DEX and/or 20 µM RU486, as indicated in figure legends, 24 hrs after transfection, after which cells were washed with pre-warmed PBS and lysed with 100 µl lysis buffer (PE Biosystems). Harvested cells were frozen at –20°C overnight. Luciferase and β-galactosidase activities were determined using the luciferase assay kit (Promega) and the Galacto-star assay kit (PE Biosystems) according to the instructions of the manufacturers. Light emission was measured in a luminoskan plate reader (Labsystems) and luciferase values were normalized for β–galactosidase activity and plotted as a percentage of the average control. EC_50_ values were determined by fitting a dose response curve.

### Site-directed mutagenesis

By means of site-directed mutagenesis (Stratagene Quick Mutagenesis kit), specific point mutations were introduced at the regulatory sites for C/EBPβ (P5), HNF3α (P4), and DBP (P3) of the rat *Cbg*295Luc promoter, by using the following primer sets: P3MUT1 5′ GTTAATTTCTAGAATTGCATGTTTACC 3′(new XbaI), P3MUT2 5′ GTTAATTTGCAGAATTCCATGTTTACC 3′ (new EcoRI), P3MUT3 5′ GTTAATTTGCAGGATCCCATGTTTACC 3′ (new BamHI), P4MUT1 5′ CAGCCACACTTAGATCTTAAAAATAAAACTAGGG 3′(new BglII), P4MUT2 5′ CAGCCACACTTCTAGATTAAAAATAAAACTAGGG 3′(new XbaI), P5MUT1 5′ CCACAAATACCATGGCAACTCCAGC 3′ (new NcoI), P5MUT2 5′ CAAATACTGACGCGGATCCAGCCACAC 3′ (new BamHI/Gsu I deleted).

Mutations were designed to create or remove restriction sites and corresponding mutated plasmid clones were sequence-verified. Since the sequence verification failed for the P4MUT1 mutation, this mutant was not further evaluated in transfection studies. All other sequence approved mutated plasmid clones were used in transfection studies.

The rat *Cbg*295Luc promoter reporter constructs (360 ng plamid DNA) with various mutations at the regulatory sites for C/EBPβ (P5), HNF3α (P4), and DBP (P3) were subsequently transiently transfected into COS-7 cells, which were plated as described for BWTG3 cells in 24-well culture plates. The transiently transfected cells were treated with 10 µM DEX or vehicle control (0.1% EtOH) for 24 hrs. Luciferase activity was compared relative to the corresponding control and normalized to protein levels.

### Chromatin immunoprecipitation assay

Twenty-four hrs after seeding, the culture medium of BWTG3 cells was replaced with DMEM, supplemented with 10% charcoal-stripped fetal bovine serum (FBS), penicillin-streptomycin (40 U/ml) and L-glutamine (30 g/l), and incubated for 24 hrs. The following day cells were either treated with 10 µM DEX or vehicle alone for 2 hrs and were then cross-linked with formaldehyde at a final concentration of 1% for 10 min at 37°C. The cross-linking reaction was stopped by the addition of glycine to a final concentration of 0.125 M for 5 min at room temperature while shaking. Cells were washed with ice-cold PBS, harvested, resuspended in 500 µl nuclear lysis buffer (1% (w/v) SDS, 50 mM Tris-HCL, pH 8.0, 10 mM EDTA plus 1 tablet 1 x Complete Mini Protease Inhibitor Cocktail per 10 ml) and sonicated at 75% Power, for 25 cycles at 20 sec per cycle, with 20 sec intervals between pulses, using the Misonix Ultrasonic Liquid Processor. The average chromatin size after sonication was about 150–500 bp. Sonicated chromatin was then centrifuged for 10 min at 15 000 g at 4°C and chromatin-containing supernatant was transferred to a clean micro centrifuge tube. After quantification by spectrophotometry, 100 µg sonicated chromatin was diluted with IP dilution buffer (0.01% (w/v) SDS, 20 mM Tris-HCL, pH 8.0, 1.1% (v/v) Triton X-100, 167 mM NaCl, 1.2 mM EDTA plus 1 x Complete Mini Protease Inhibitor Cocktail at 1 tablet per 10 ml), followed by pre-clearing for 1 hr with 20 µl 50∶50 (v/v) pre-blocked Protein A/G PLUS beads (sc-2003, Santa Cruz Biotechnology, USA) on a rotating wheel at 4°C. Pre-cleared chromatin was incubated with 2 µg primary antibody (anti-GR (GR-H300 sc-8992), anti-C/EBPβ (C/EBβ-C19 sc-150), or anti-IgG antibody (sc-2027), all from Santa Cruz Biotechnology) overnight at 4°C on a rotating wheel. The following day, the mixture was incubated with 40 µl 50∶50 (v/v) pre-blocked Protein A/G PLUS beads on a rotating wheel for 6 hrs at 4°C. The samples were then centrifuged at 5000 g for 1 min at 4°C and the pellet was washed three times with 1 ml wash buffers with increasing salt concentration (150 mM –500 mM NaCl) and followed by three washes with 1 ml TE buffer. After washing, the pellet was resuspended in 300 µl elution buffer (1% (w/v) SDS and 100 mM NaHCO_3_). Cross-linking was reversed by the addition of NaCl at a final concentration of 300 nM, followed by incubation overnight at 65°C. Thereafter a further incubation at 45°C for 1 hr in the presence of 15 nM EDTA, 125 nM Tris-HCL and 60 ng/ml proteinase K (Roche Applied Science) was performed. Chromatin DNA was purified using NucleoSpin Extract II nucleic acid purification kit (Macherey-Nagel). Purified chromatin DNA was amplified by semi-quantitative real-time PCR using primers, spanning (1) the regions P5 to P1 FWD:5′-CCCTGCCAGGTGGCACAGG-3′; REV:5′-GGAGAGGGGCAGTGGCCTTC-3′ of the m*Cbg* promoter and (2) a non-specific region within the m*Cbg* promoter (position −141/−35), FWD:5′- AGGGGGTGGGGACCACCAAA-3′; REV:5′- AAGGCTTCGGGGAGACTCCTACTA-3′. ChIP results were normalised as described by Aparicio and co-workers [41]. Briefly, Ct values for amplification of purified IP chromatin DNA from the regions of interest were normalized to Ct values from a control non-specific region within the m*Cbg* promoter, as well as normalized input. DEX treatment did not affect recruitment of transcription factors to the non-specific region (data not shown).

In re-ChIP experiments, chromatin-antibody complexes were first eluted in 100 µl elution buffer containing 10 mM dithiothreitol and 1% SDS at 37°C for 30 min whilst shaking. The eluted samples were diluted 20 times with IP dilution buffer and subjected again to the ChIP procedure with an antibody specific for C/EBPβ (C-19 sc-150 Santa Cruz Biotechnology). The first immunoprecipitation with an anti-GR antibody served as internal control. Ct values from re-ChIP results were normalized to Ct values from IgG vehicle control, which was set as 1.

### Small interference RNA (siRNA) transfections

BWTG3 cells plated at a density of 1 × 10^5^ cells/well in a 12-well culture plate were transfected with 10 nM validated C/EBPβ siRNA, which consist of four different validated siRNA oligo’s (Qiagen Flexitube GeneSolution cat# GS12608) directed against the mouse C/EBPβ, or validated non-silencing scrambled sequence control (NSC) siRNA (cat#1027310) (Qiagen), using HiPerfect transfection reagent (Qiagen) as per the manufacturer’s instructions. Briefly, C/EBPβ or NSC siRNA was diluted in pre-warmed Optimem medium (Invitrogen Gibco-BRL Life Technologies) to which 4.5 µl transfection reagent was added. Cells were incubated for 24 hrs before being treated with 1 nM DEX for 8 hrs or vehicle control in unsupplemented medium, after which RNA was harvested and CBG gene expression analyzed by semi-quantitative real time PCR. In addition, lysates were prepared for Western blot analysis using a CBG-specific antibody (ab107368) from Abcam.

For ChIP, where C/EBPβ protein expression was silenced, BWTG3 cells were plated in 10-cm tissue culture dishes at a density of 1×10^7^ cells per dish. Twenty-four hours after seeding, cells were transfected as described above only with amounts adjusted for the larger seeding vessel. Briefly, 150 nM validated C/EBPβ siRNA directed against the mouse C/EBPβ, or validated NSC siRNA were incubated together with pre-warmed Optimem medium and 67.5 µl HiPerfect transfection reagent as per the manufacturer’s instructions whereafter it was added to the cells. Twenty-four hours after siRNA transfection the ChIP protocol was followed as described earlier. Briefly, Ct values for amplification of purified IP chromatin DNA from the regions of interest were normalized to Ct values from a control non-specific region within the m*Cbg* promoter, normalized input, and NSC vehicle control, which was set as 1. Protein knockdown for all experiments was verified by Western blot analysis.

### Data and statistical analysis

Data is expressed as the mean ± SEM for triplicate values of each experiment and analyzed by ANOVA followed by the Dunnett’s, or Bonferroni’s multiple comparison’s posttest (p<0.001***; p<0.01**; p<0.05*; p>0.5 not significant) or, when comparing two groups, only one-tailed paired t-test was used. Each experiment was repeated at least twice. Graph Pad Prism® was used for graphical representation and statistical analysis.

## Results

### Regulation of CBG mRNA by physical and psychological stressors in rats

**Figure 2:**
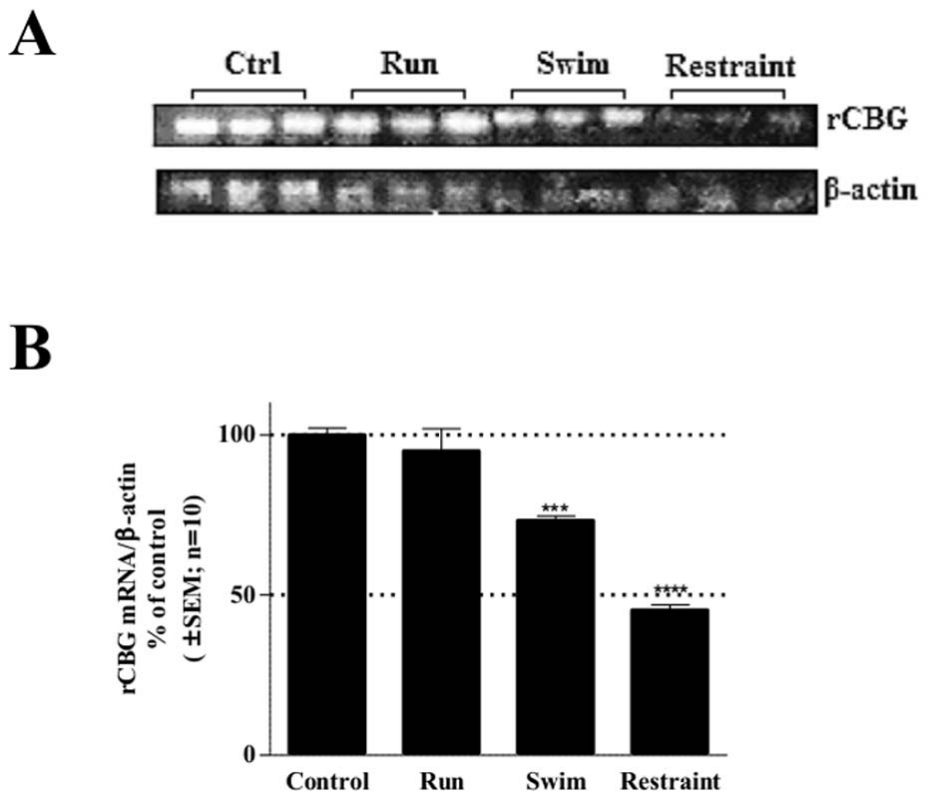
Regulation of rat CBG mRNA levels by physiological stressors. Adult male Wistar rats were subjected to voluntary running (Run), involuntary swimming (Swim) or restraint (Restraint) treatment for ten days (see Experimental Procedures). Rats were sacrificed and livers removed for RNA isolation, which was analyzed by Northern blotting. (A) Representative Northern blot of CBG and β-actin mRNA levels. (B) Quantification of CBG mRNA expression levels normalized to liver β-actin mRNA levels. Statistical analysis was done relative to the corresponding control rats, using one-way ANOVA followed by Dunnett’s multiple comparison’s post-test (***: p<0.001; ****:p<0.0001).

Numerous reports have indicated that CBG expression is influenced by both physical and psychological stressors [21], [42]. With this in mind, adult male Wistar rats were subjected to a mild psychological stressor, namely, restraint, and two physical stressors of diverse intensity, namely, voluntary running (mild stressor) or involuntary swimming (severe stressor). Intermittent stress exposure continued for ten days after which rats were sacrificed and livers removed for RNA isolation (Figure 2). Of all the stressful events, restraint affected CBG mRNA expression most significantly (p<0.0001) with a 55% reduction, followed by involuntary swimming (p<0.001) with a 27% reduction. In contrast, voluntary running had little effect on CBG mRNA expression. These findings are in agreement with the literature which has reported a decrease in CBG expression due to stress [4], [20], [21], [42].

### Regulation of CBG mRNA and protein levels by DEX in hepatic cell lines

**Figure 3:**
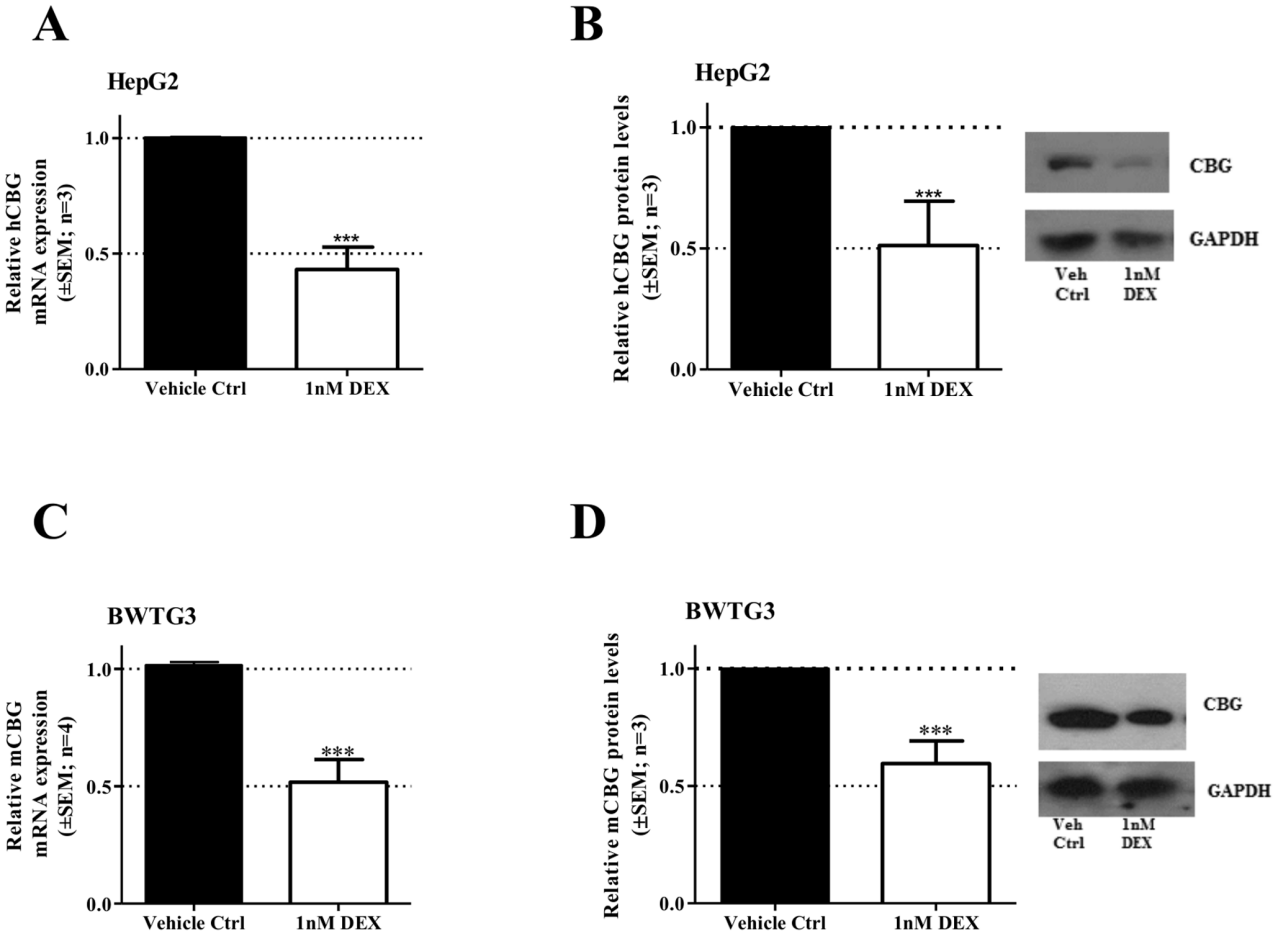
DEX treatment down-regulates CBG mRNA and protein levels in hepatoma cell lines. The effect of DEX on CBG mRNA and protein levels was investigated in a human hepatoma cell line, HepG2 (A&B) and in a mouse hepatoma cell line, BWTG3 (C&D). Both cell lines (HepG2 & BWTG3) were incubated with vehicle control (0.1% EtOH) or 1 nM DEX for 8 hrs to determine CBG mRNA (A&C) and protein expression (B&D). Total RNA was isolated and reversed transcribed to cDNA. Real-time quantitative PCR (qPCR) was performed to determine the mRNA expression levels of CBG and internal standards (18S for HepG2 and GAPDH for BWTG3 cells). CBG protein expression was analyzed by means of Western blotting. GAPDH protein expression was used as loading control. Statistical analysis was done relative to the corresponding vehicle control (0.1% EtOH), using students unpaired *t*-test (*: p<0.05; **: p<0.01; ***:p<0.001).

During stress endogenous GCs are released, which are believed to be the mediators responsible for the *in vivo* decrease in CBG expression [30]. In addition, the potent GC, DEX, has been shown to decrease CBG mRNA in mice [30]. Thus, to establish whether the effect observed by us, and others, *in vivo* regarding CBG expression is also seen *in vitro*, human and mouse hepatoma cell lines, HepG2 and BWTG3, respectively, were treated with DEX. Both CBG mRNA and protein levels were evaluated (Figure 3). The results show that DEX at 1 nM significantly (p<0.05) decreased CBG mRNA and protein expression in both cell lines tested. As fold decrease in CBG expression was similar for both mRNA and protein, this may suggest that regulation occurs primarily at the transcriptional level. Our results in HepG2 cells are in contrast to a previous study showing no effect of 1 µM DEX at mRNA and on protein levels [43].

### The GR is involved in DEX-mediated repression of CBG expression

**Figure 4:**
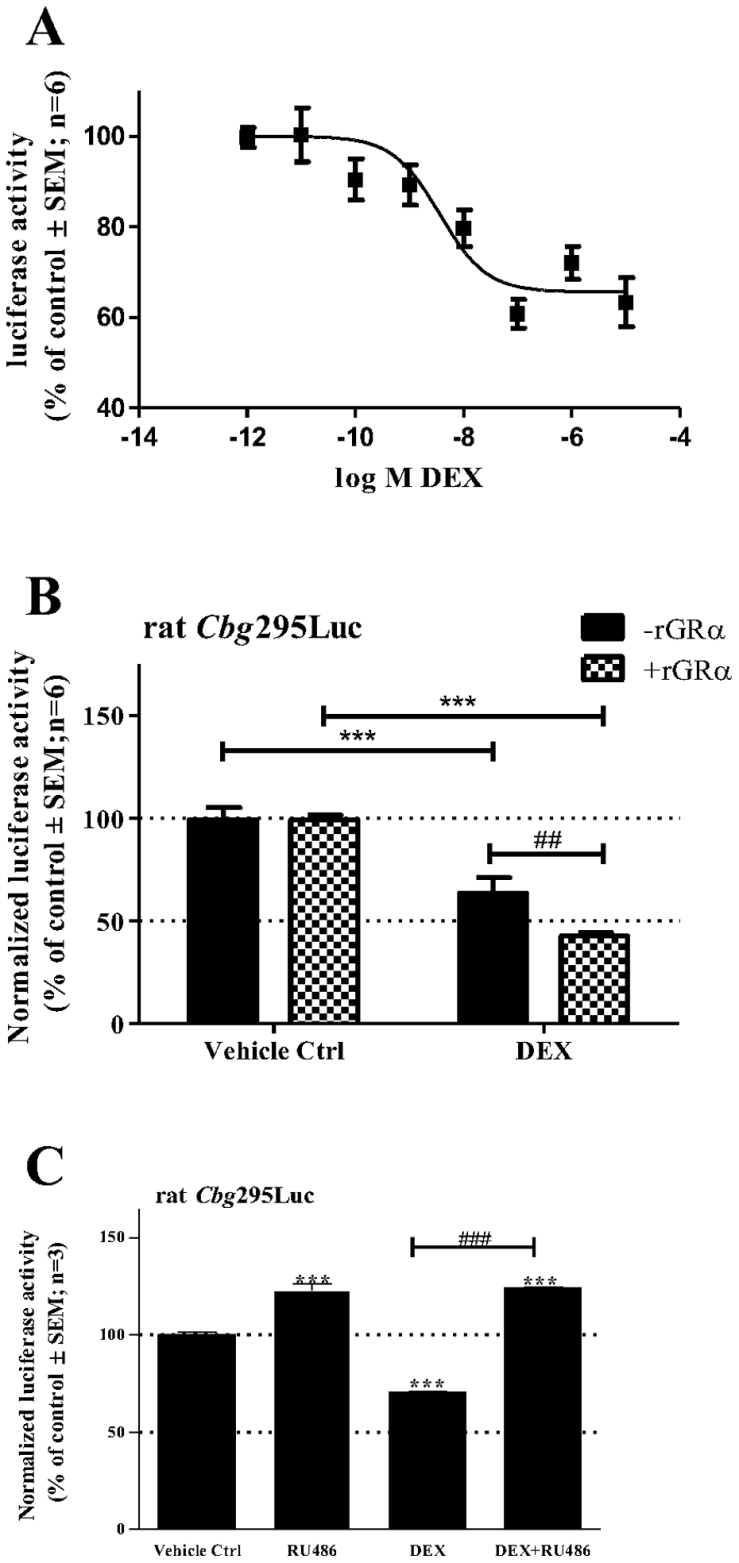
DEX-mediated repression of the rat *Cbg*295Luc promoter reporter construct is dependent on GR. (A) DEX represses the rat Cbg295Luc promoter reporter construct in a dose dependent manner in BWTG3 cells. The cells were transiently transfected with rat *Cbg*295Luc and with a β-galactosidase expression plasmid (pPGKβGopbA) to monitor for transfection efficiency. Twenty-four hrs after transfection, cells were treated with increasing concentrations of DEX and incubated for 24 hrs. Luciferase values were normalized for β–galactosidase activity and plotted as a percentage of the average control. EC_50_ values were determined by fitting a dose response curve. (B) Effect of co-transfected GR expression vector on transrepression of the rat *Cbg*295Luc promoter reporter construct by DEX was investigated in BWTG3 cells. The cells were transiently transfected with rat *Cbg*295Luc, with or without the rat GRα expression vector, pSVGR1. In addition, a β-galactosidase expression plasmid (pPGKβGopbA) was co-transfected to monitor for transfection efficiency. Twenty-four hrs after transfection, cells were treated with 1 µM DEX or vehicle control (0.1% EtOH) and incubated for 24 hrs. Luciferase values were normalized to β–galactosidase and values plotted as a percentage of the average vehicle control. Statistical analysis was done to (i) compare values in presence of DEX relative to the corresponding control using one-way ANOVA followed by Dunnett’s multiple comparison’s posttest (***: p<0.001) and to (ii) compare values without GR (–rGRα) to values with co-transfected GR (+rGRα) using two-way ANOVA followed by Bonferonni’s multiple comparison’s posttest (##: p<0.01). (B) Effect of the glucocorticoid antagonist, RU486, on transrepression was determined by transiently transfecting BWTG3 cells with the rat *Cbg*295Luc promoter reporter. Twenty-four hrs after transfection cells were treated with 1 µM DEX and/or 20 µM RU486, as indicated. Luciferase values were normalized to β–galactosidase and values are plotted as a percentage of the average vehicle control. Statistical analysis was done to (i) compare values in presence of test compounds relative to vehicle control (0.1% EtOH) (***: p<0.001) and to (ii) compare values of each compound tested relative to the combined treatment of DEX and RU486 (DEX+RU486) (###: p<0.001), using one-way ANOVA followed by Bonferonni’s multiple comparison’s posttest comparing all columns.

Once it was established that endogenous CBG expression is repressed by the synthetic glucocorticoid, DEX, we next tested whether the r*Cbg*295Luc promoter reporter construct would behave similarly. BWTG3 cells were transiently transfected with the r*Cbg*295Luc promoter reporter construct in the presence of increasing DEX concentrations (Figure 4A). The dose-response curve indicates that DEX represses the r*Cbg*295Luc promoter reporter construct with an EC_50_ value of 3.6 nM. Although repression at 1 nM is thus less than seen for the endogenous r*Cbg* gene (Figure 3C) at around 1 µM DEX, maximal repression is observed.”

Having established that DEX can repress the r*Cbg*295Luc promoter reporter construct in a dose-dependent manner, we next tested whether this transrepression potential is dependent on the glucocorticoid receptor (GR) as glucocorticoids act *via* this ligand-activated steroid receptor [18]. BWTG3 cells were transiently transfected with the r*Cbg*295Luc promoter reporter construct in the presence or absence of the transfected rGRα expression vector (Figure 4B). Co-transfection with rGRα in BWTG3 cells significantly (p<0.01) increased DEX-induced (1 µM) repression of r*Cbg* promoter activity (Figure 4B). GR dependence of DEX-mediated inhibition of r*Cbg* promoter activity was further confirmed as co-treatment with the GR antagonist, RU486, significantly (p<0.001) abolished DEX-mediated repression of r*Cbg* promoter activity (Figure 4C). These two results strongly suggest that DEX-induced repression of r*Cbg* promoter activity acts through the GR and is in agreement with results obtained by Cole and co-workers [30].

### Delineation of DEX responsiveness within the *rCbg* proximal promoter reporter construct

**Figure 5:**
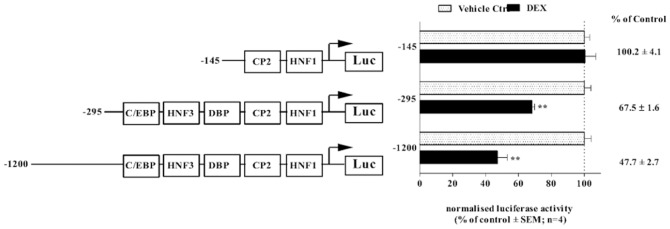
Delineation of DEX-mediated repression of the rat *Cbg* promoter. The rat *Cbg*1200Luc, rat *Cbg*295Luc and rat *Cbg*l45Luc promoter reporter constructs were transiently transfected into BWTG3 cells. A β-galactosidase expression plasmid (pPGKβGopbA) was co-transfected to monitor for transfection efficiency. Twenty-four hrs after transfection, cells were treated with 10 µM DEX or vehicle control (0.1% EtOH) and incubated for 24 hrs. Luciferase activity was determined and normalized to β–galactosidase activity. Results are plotted as a percentage of the average vehicle control (0.1% EtOH), which was set as 100%. Statistical analysis was done to compare treatments in the presence of DEX relative to vehicle control (0.1% EtOH), using one-way ANOVA followed by Dunnett’s multiple comparison’s posttest (**: p<0.01).

Although we and others have shown involvement of the GR in DEX-induced repression of CBG, no consensus binding element(s) for the GR could be detected in the *rCbg* promoter by transcription factor motif analysis. Thus, next, we wanted to determine which region of the proximal *Cbg* promoter plays a role in GR-mediated modulation of CBG expression. BWTG3 cells were therefore transiently transfected with the full length *rCbg* proximal promoter reporter construct (rat *Cbg*1200Luc), as well as with the truncated *rCbg* promoter reporter constructs (rat *Cbg*295Luc and rat *Cbg*145Luc) (Figure 5). The rat *Cbg*295Luc promoter reporter construct contains the five protein-binding sites (P1–P5), identified by DNase I foot printing, which resemble recognition sequences for hepatocyte nuclear factor-1 (HNF1β), CCAAT-binding protein-2 (CP-2), D-site binding protein (DBP), hepatocyte nuclear factor-3 (HNF3α) and CAAT/enhancer-binding protein (C/EBPβ), whereas the rat *Cbg*145Luc promoter reporter construct contains only HNF1β and CP-2 [25].

The glucocorticoid, DEX, at 1 µM, significantly transrepressed (p<0.01) the transiently transfected rat *Cbg*1200Luc and rat *Cbg*295Luc promoter reporter constructs, as was observed with the endogenous gene (Figure 3). In contrast, DEX had no effect on the rat *Cbg*145Luc promoter reporter activity, which contained only the HNF1β and CP-2 binding sites, suggesting that these two sites are not important for GC-mediated repression of *Cbg* promoter activity (Figure 5).

### GC responsiveness is abolished, when the C/EBPβ site in the r*Cbg* proximal promoter reporter construct is mutated

**Figure 6:**
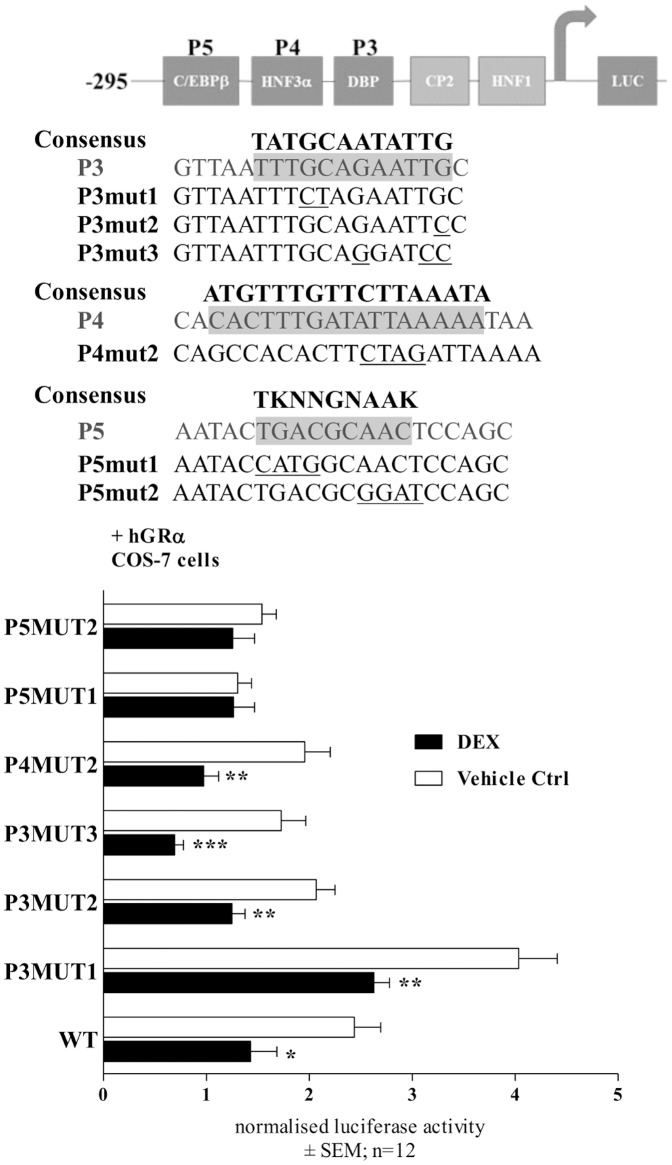
DEX responsiveness is abolished when the C/EBPβ site is mutated. The rat *Cbg*295Luc promoter reporter construct with various mutations at regulatory sites C/EBPβ (P5), HNF3 (P4), and DBP (P3) were transiently transfected into COS-7 cells. Transfected cells were treated with 10 µM DEX or vehicle control (0.1% EtOH) for 24 hrs. Luciferase activity was measured and normalized to protein concentrations. Results were compared relative to the corresponding vehicle control (0.1% EtOH). For statistical analysis two-way ANOVA was done with Bonferroni’s multiple comparison as post-test to compare DEX-induced treatments with each corresponding control (0.1% EtOH) (*: p<0.05; **: p<0.01; ***: p<0.001).

Following the delineation study of the *Cbg* promoter (Figure 5), which identified the region containing the binding elements for C/EBPβ (P5), HNF3α (P4), and DBP (P3) as being important for DEX-mediated repression of CBG, site-directed mutagenesis of the above mentioned regulatory binding elements was performed. DEX (10 µM) was still able to significantly down-regulate *Cbg* promoter activity, when HNF3α (P4) and DBP (P3) sites were mutated (Figure 6). In contrast, mutations of the C/EBPβ-binding site resulted in a loss of DEX-induced repression (Figure 6), suggesting that the C/EBPβ *cis*-regulatory site is important for DEX-mediated regulation of CBG.

### The GR is recruited to the C/EBPβ cis-regulatory site in the *Cbg* gene promoter

Having shown that the GR is required for DEX-mediated inhibition of CBG (Figure 4) and that mutating the C/EBPβ regulatory site influences this repression (Figure 6), we next wanted to investigate whether the GR occupies the endogenous m*Cbg* promoter by means of a ChIP assay.

**Figure 7:**
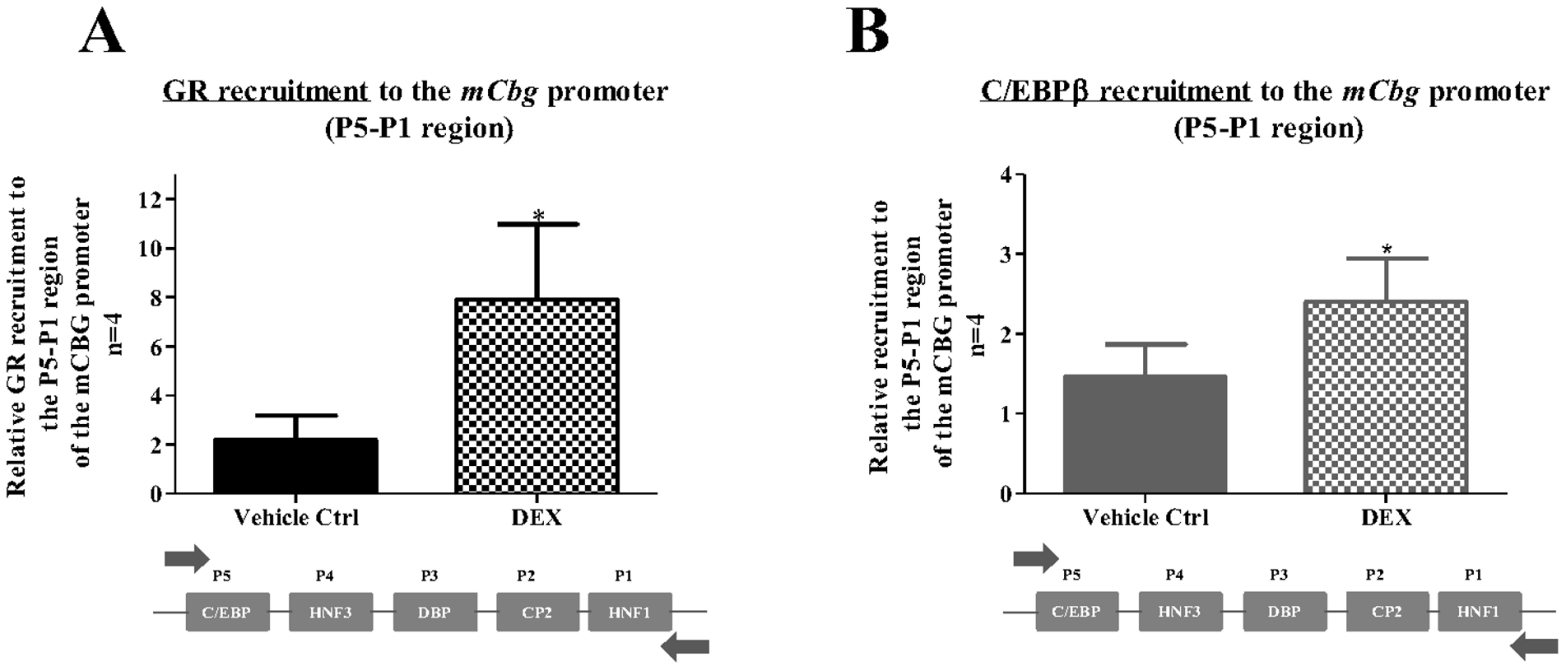
The GR is recruited to the mouse *Cbg* promoter in response to DEX. BWTG3 cells were treated with 10 µM DEX for 2 hrs followed by ChIP assay, as described in the Material and Methods. Occupancy of GR protein on the endogenous mouse *Cbg* promoter was detected using primers encompassing the regions defined as P5 to P1 (A). In addition, C/EBPβ recruitment onto the m*Cbg* promoter encompassing regions P5 to P1 was determined (B). Co-immunoprecipitated DNA fragments and input DNA were analyzed by qPCR and results shown are normalized to a non-specific region of the m*Cbg* promoter, input and the vehicle control (0.1% EtOH) of a non-specific region, which was set as 1. Statistical analysis was done to compare recruitment of the GR in response to DEX relative to vehicle control (0.1% EtOH), using student’s paired *t*-test (*: p<0.05; ***:p<0.001).

In response to DEX treatment, significant enrichment of GR occurred at the endogenous m*Cbg* promoter (P5 to P1) relative to a control non-specific region within the *mCbg* promoter (Figure 7A). This area of the m*Cbg* promoter contains the C/EBPβ-binding element (P5). Furthermore, significant DEX-induced C/EBPβ recruitment to the *Cbg* promoter was also observed (Figure 7B) substantiating for the first time that C/EBPβ does indeed bind to the *Cbg* proximal promoter. These results, taken together with the loss of DEX-induced repression of the *Cbg* promoter when the C/EBPβ *cis*-regulatory element was mutated (Figure 6), suggests that the GR is binding to the C/EBPβ-binding element (P5).

### C/EBPβ knockdown attenuates DEX-mediated CBG mRNA and protein repression and GR recruitment to the *Cbg* promoter

**Figure 8:**
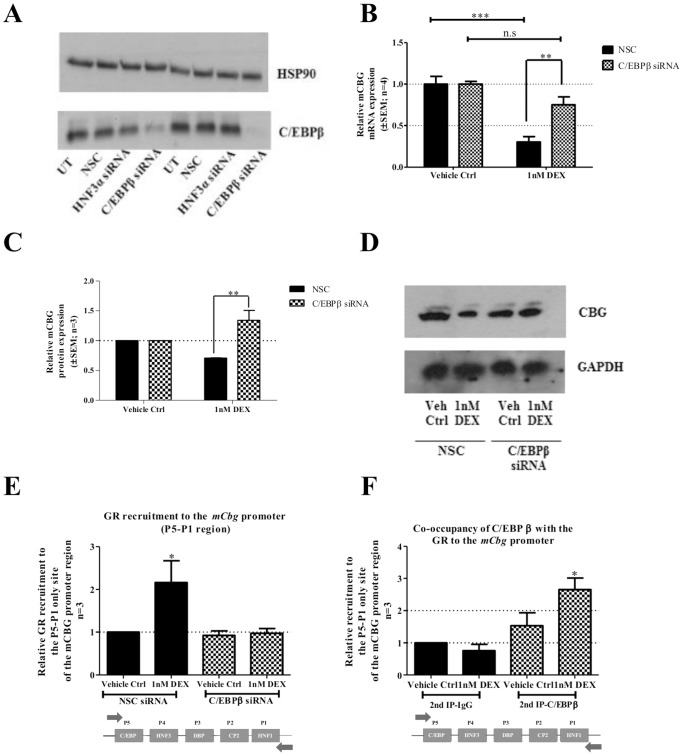
Decrease of C/EBPβ protein expression attenuates DEX-induced repression of CBG mRNA and protein levels and reduces GR recruitment to the mouse *Cbg* promoter. To determine CBG mRNA and protein expression in response to decreased C/EBPβ protein expression, BWTG3 cells were transfected with non-specific siRNA (NSC) or with siRNA specific for the mouse C/EBPβ. As a control for C/EBPβ knockdown, total protein from untransfected (UT) BWTG3 cells, as well as from BWTG3 cells transfected with NSC or C/EBPβ siRNA, was harvested to perform Western blotting. A representative blot of two independent experiments is shown (A). Cells were treated with 1 nM DEX or vehicle control (0.1% EtOH) for 8 hrs, 24 hrs after transfection. (B) Relative CBG mRNA expression levels were measured by qPCR and normalized to relative GAPDH. Relative fold-expression of CBG mRNA levels were normalized to respective vehicle control (0.1% EtOH) which was set as 1. (C) CBG protein expression levels were analyzed by means of Western blotting, quantified and normalized to relative GAPDH expression. Relative CBG protein expression were normalized to the respective vehicle control (0.1% EtOH), which was set as 1. (D) A representative Western blot of CBG protein expression is shown. For (B&C) statistical analysis was done to compare CBG mRNA and protein expression in response to decreased C/EBPβ protein expression using two-way ANOVA with Bonferroni' multiple comparison as post-test (*: p<0.05; **:p<0.01; ***:p<0.001). (E) To examine the effect of decreased C/EBPβ protein expression on GR recruitment to the mouse *Cbg* promoter, BWTG3 cells were transfected with siRNA as described above. Twenty-four hrs after transfection, cells were treated with 10 µM DEX or vehicle control (0.1% EtOH) for 2 hrs followed by ChIP assay, as described in the Material and Methods. Occupancy of GR protein on the endogenous mouse *Cbg* promoter was detected using primers encompassing the regions defined as P5 to P1. Co-immunoprecipitated DNA fragments and input DNA were analyzed by qPCR and results shown are normalized to a non-specific region within the m*Cbg* promoter as well as input and expressed relative to NSC vehicle control (0.1% EtOH), which was set as 1. Statistical analysis was done to compare recruitment of the GR relative to NSC vehicle control (0.1% EtOH), using one-way ANOVA followed by Dunnett’s multiple comparison’s posttest (*: p<0.05). (F) Co-recruitment of the C/EBPβ and GR to the mouse *Cbg* promoter in response to DEX. BWTG3 cells were treated with 10 µM DEX for 2 hrs followed by re-ChIP assay, where cross-linked cell lysates were subjected to immunoprecipitation, first with an anti-GR-specific antibody (data not shown) and then with a control IgG or an anti-C/EBPβ-specific antibody (IP2). The occupancy of GR and C/EBPβ protein at the *Cbg* promoter, encompassing regions P5 to P1 were detected using primers designed against the indicated region. Co-immunoprecipitated DNA fragments and input DNA were analyzed by qPCR. Results shown were normalized to IgG for second IP as well as input and expressed relative to IgG vehicle control for the second IP (0.1% EtOH), which was set as 1. Statistical analysis was done to compare DEX-induced protein recruitment to the *Cbg* promoter relative to IgG vehicle control (0.1% EtOH), using one-way ANOVA with Dunnett’s multiple comparison as post-test (*: p<0.05).

Due to recruitment of the GR to the *Cbg* promoter in the absence of any known GR-binding element, we postulated that GR-mediated repression of CBG expression is *via* interaction with C/EBPβ through a tethering mechanism. This argument is strengthened by the observations identifying the C/EBPβ-binding element as important for DEX responsiveness (Figure 6). Knockdown studies of C/EBPβ protein were thus performed to confirm the involvement of C/EBPβ in DEX-induced repression of CBG (Figure 8). A decrease in C/EBPβ protein expression was achieved using a mixture of four validated siRNA oligo’s specific for the mouse C/EBPβ (Figure 8A).

The non-specific scrambled siRNA oligo’s (NSC) had no effect on C/EBPβ expression (Figure 8A) or DEX-induced repression of CBG mRNA and protein expression (Figure 8B, C&D). However, the siRNA specific decrease in C/EBPβ protein expression resulted in a significant (p<0.01) abrogation of DEX-induced repression of CBG mRNA and protein expression in BWTG3 cells (Figure 8B, C&D).

Furthermore, to establish whether occupancy of ligand-activated GR on the *Cbg* gene promoter is dependent on C/EBPβ, ChIP assays were performed with cells transfected with C/EBPβ-specific siRNA oligo’s to decrease C/EBPβ protein expression. As shown in Figure 8E, when C/EBPβ protein expression was decreased, GR recruitment to the *Cbg* gene promoter in the presence of DEX was significantly (p<0.05) diminished.

The results obtained with the C/EBPβ knockdown experiments (Figure 8), together with the site-directed mutagenesis experiments (Figure 6) and the ChIP results presented in Figure 7 strongly suggest that DEX-induced repression of CBG involves C/EBPβ.

### The GR together with C/EBPβ occupies the C/EBPβ cis-regulatory element of the *Cbg* promoter

Ligand-activated GR has been shown to interact with C/EBPβ [35]. To assess whether these transcription factors co-occupy the *Cbg* promoter in response to DEX, a re-ChIP assay was performed using an anti-GR antibody for the first immunoprecipitation (IP1), followed by the second immunoprecipitation (IP2) step using an anti-C/EBPβ-specific antibody. The results show that both GR and C/EBPβ are present in a complex and, in response to DEX treatment are recruited to the *Cbg* promoter encompassing regions P1–P5 (Figure 8F).

## Discussion

CBG has been extensively characterized as a carrier protein [4], [7], [14] and as a reservoir of endogenous GCs [19]. Because CBG levels directly affect GC bioavailability and consequently GC signaling in homeostatic stress responses, evaluation of factors that modulate CBG levels are of interest.

The current study focused on the molecular mechanism of GC-mediated inhibition of CBG expression. GCs are the major hormone secreted during stress and we show that a variety of stressors, both physical and psychological, influenced CBG mRNA levels *in vivo* (Figure 2). In addition, we found that the severity of the stressor modulated the amplitude of the response. Specifically, voluntary running, considered the least stressful intervention, showed little effect on hepatic CBG mRNA expression, while both of the involuntary stressors, swimming (physical) and restraint (psychological), resulted in a significant inhibition of CBG mRNA. The psychological stress induced through restraint of the rats, considered the most stressful event, showed the highest inhibition (55%) of rat CBG mRNA expression. These results suggest (i) that the severity of the stressor influences the degree of CBG expression modulation, and (ii) that stress affects CBG expression at a transcriptional level. This is in agreement with earlier reports for both rats and humans [7], [13], [15], [17]. During stress endogenous GCs are released from the adrenals due to the activation of the hypothalamic-pituitary-adrenal (HPA) axis and the resulting increased circulating levels of GCs are believed to be responsible for the inhibition of CBG expression [7]. In support of the fact that GCs directly regulate CBG expression, we observed that treatment with the potent GC, DEX, on its own resulted in a significant decrease in both CBG mRNA and protein levels in hepatoma cell lines (Figure 3). This is in agreement with a previous study showing that in rats treated with DEX for 48 hrs, hepatic CBG mRNA levels and CBG serum protein concentrations were significantly decreased [31].

Having shown that stress, and specifically GCs, repress CBG levels we determined that the effect is mediated by the GR, the ligand-activated transcription factor required for the intracellular effects of GCs (Figure 4) [44]. Specifically, we showed that overexpression of GR potentiated the DEX-induced repression of CBG (Figure 4B), whereas co-treatment with the GR antagonist, RU486, attenuated DEX-mediated repression of a *Cbg* promoter construct (Figure 4C). Furthermore, recruitment of the GR to the *Cbg* gene promoter increased in response to DEX (Figure 7A). These results suggest that the GR plays an important role in GC-mediated repression of CBG and is supported by a previous study showing that mice without a functional GR were resistant to DEX-mediated repression of hepatic CBG mRNA [30].

Despite the fact that the proximal promoters of the CBG gene have been cloned and that five putative transcription factor-binding sites (P1–P5) within the sequence have been identified (Figure 1) [25], the current study is the first to investigate the *cis*-acting elements involved in GC regulation. Initial experiments delineating the DEX-responsiveness of the *Cbg* promoter established that the region encompassing −145 bp from the transcription start site, which contains P1 and P2, was unresponsive to DEX treatment (Figure 5). The *cis*-regulatory elements associated with P1 and P2 have previously been identified as HNF1β and CP2 [29], respectively, and, although the current study established that these *cis*-regulatory elements are not important for DEX-mediated repression of CBG, the region was previously shown to be transcriptionally active and is probably required for minimal promoter activity [25].

As the region −295 bp from the transcription start site, which encompasses P1–P5, resulted in DEX-induced repression of the *Cbg* promoter (Figure 5) and recruited GR (Figure 7A), this left the binding sites P3–P5, as possible candidates for DEX-mediated repression of CBG expression. P3–P5 have been suggested to resemble recognition sequences for DBP, HNF3α and C/EBPβ, respectively [25], although this has not yet been unequivocally demonstrated experimentally. Site-directed mutagenesis of C/EBPβ (−216/−236), HNF3α (−189/−214), and DBP (−148/−170) binding sites in the *Cbg* promoter narrowed down the candidate *cis*-acting elements and identified the C/EBPβ *cis*-regulatory site, but not HNF3α or DBP, as important in DEX-induced repression of CBG (Figure 6). In addition, a decrease in C/EBPβ protein expression by siRNA resulted in the attenuation of DEX-induced repression of CBG mRNA and protein expression in BWTG3 cells (Figure 8B, C&D), as well as GR recruitment to the *Cbg* promoter (Figure 8E).

In further support of C/EBPβ’s involvement in GC-mediated repression of CBG, we also show that the GR together with C/EBPβ co-exist in a complex that occupies the C/EBPβ *cis*-regulatory position (Figure 8F). In addition, ChIP results (Figure 7B) also suggest that ligand-activated GR does not disturb C/EBPβ occupancy, thereby inhibiting transcription as reported for IL-1β [45]. Rather, CBG inhibition appears to resemble COX-2 repression by GCs, which requires C/EBPβ and GR to form a protein-protein interaction in occupying the COX-2 promoter [46]. The data in this study would suggest that the molecular mechanism of GR-induced repression of CBG is similar to that proposed for COX-2 and many other pro-inflammatory genes inhibited by GCs. This would entail that the ligand-activated GR physically interacts with C/EBPβ, possibly *via* a tethering mechanism whereby both transcription factors are present on the *Cbg* promoter. Physical interaction of the GR with C/EBPβ has been described for genes that are positively and negatively regulated by GCs, most of which are involved in inflammation [35], [46]–[49]. Further support for a tethering mechanism comes from previous work in our laboratory, indicating that that a GR monomer rather than a GR dimer is involved in DEX-mediated repression of CBG [32], [33].

C/EBPβ is a ubiquitously expressed transcription factor involved in the regulation of numerous cellular responses and plays an important role in regulating liver function [50]–[52]. C/EBPβ is especially known to be an important regulator of the acute phase response (APR) [52]. It modulates the expression of various acute phase proteins (APPs), such as α1-acid glycoprotein and haptoglobin [53]–[55], as well as modulating the expression of acute phase cytokines, all of which, like CBG, contain binding motifs for C/EBPβ within their promoters [56], [57]. The APR is the first response to various stressors, such as injury, bacterial infection or systemic inflammation, and is activated by inflammatory mediators, such as tumor necrosis factor alpha (TNFα), IL-1β, IL-6, and GCs. C/EBPβ is transcriptionally and post-translationally activated by these early inflammatory stimuli all of which contribute to the activation of the APR [52], [58]. A number of APPs are synergistically regulated by GCs and C/EBPβ [59]. Positive APPs, like α1 acid-glycoprotein, C-reactive protein (CRP), and serum amyloid A (SAA), are known to be regulated by GCs presumably through protein-protein interaction of the GR with C/EBPβ although the exact molecular mechanism has not been established for all APPs mentioned [58]–[63]. GC activation of CRP and SAA was reported to be inhibited, when C/EBPβ protein expression was decreased by siRNA. The authors, however, did not investigate any possible tethering mechanism of ligand-activated GR and C/EBPβ [59]. They did, however, show increased C/EBPβ binding to DNA in response to GC treatment as determined by EMSA’s [59]. This finding is in agreement with the observation made in the present study of C/EBPβ’s recruitment to the *Cbg* promoter in response to DEX (Figure 7B). Furthermore, a recent study identified C/EBPβ, to be important in GR signaling especially in the liver, as most GR binding to DNA occurs at accessible chromatin sites preoccupied by C/EBPβ [36].

In conclusion, physiological stress or intake of exogenous GCs decreases CBG levels, which have a direct effect on GC bioavailability. It is well established that GCs negatively affect their own carrier protein, and earlier studies suggested that GC-mediated downregulation of CBG occurs at a transcriptional level *via* the GR, in agreement with the findings of the present study. In addition, cumulative evidence presented here strongly suggests that ligand-activated GR tethers to C/EBPβ, which results in GC-mediated repression of CBG gene expression. Lower CBG levels have been linked to obesity and insulin resistance, as it has been shown to affect fat accumulation and muscle development probably due to an increase in circulating GCs [4], [64]–[69], although hypocortisolism has also been associated with CBG deficiency attributed to CBG functioning as a reservoir for endogenous glucocorticoids [70], [71]. Nonetheless, chronic stress or inflammation would affect CBG levels thereby directly influencing GC signaling. This study has deciphered the molecular mechanism whereby GCs influence CBG transcription levels.
